# 

**Published:** 1917-03

**Authors:** 


### ANNOUNCEMENTS

TENNESSEE STATE DENTAL ASSOCIATION. Golden Jubilee Session at Memphis June 19-22, 1917. The sessions and exhibits will be held in the same building —the Scottish Rites Temple—-one-half block from the dental college, just outside the industrial center of the city and within a block of two lines of street cars, running out from the hotel district. The audience room of the Temple is all but sound proof, having a line of class rooms surrounding it. The room is cooled by gentle breezes floating over tons of ice into the auditorium and as it becomes warmed and rises to the dome it is there drawn off by large suction fans. Surrounding this auditorium on two sides are the class rooms, at the back of it is a large stage and at the front there is a rest room the like of which is not equalled in any of the clubs in the city.

The exhibits of the Dental Manufacturers Club, which includes forty of the better class supply houses, will be in the large hall, a space equal to the auditorium and the stage combined. The small exhibitors will show their wares in the side class rooms. From the hotel there will be a line of automobiles running to the Temple every morning in ample time for the meeting, giving plenty of time to look over the exhibits. These automobiles will carry the visitors to the temple for the morning session and evening session. Luncheon will be served in the building from the temple kitchen—somewhat in the style of a cafetiere. After luncheon, until the gavel falls, the audience will have the benefit of that rest room and the beautiful little park just across the boulevard—a splendid place to take the fresh air.

There will be but one night session and the public entertainment that night, in all probability, will be given by Dr. Charles Mayo, of Rochester, Minn. Dr. Mayo has not positively promised but the Program Committee is expecting him to be with us. The Program Committee has secured for the regular session work several other "high brow" entertainers, who will bring their magic lanterns and moving picture shows with them.

It being the "Jubilee" year it is hoped that the gentlemen will bring their wives or sweethearts as we propose to care for the social side of life as well as the intellectual. For the visitors there will be a moonlight boat ride on the great "father of waters." There will be an automobile drive covering the beautiful boulevards, driveways and parks of the city. In that, we will have a surprise for all those who have not covered the ground. But few people know of the beautiful driveways, palatial residences and artistic park system of Memphis. Of course the banquet will be a prominent feature, with the flowery speeches that belong to the orators of the South. For the visiting ladies, while the men are hard at work, there will be other entertainments such as luncheons, drives, shows, etc. This will be managed by a committee of ladies. So, the social as well as the professional entertainment will belong to this Golden Jubilee meeting. Through the journals we expect to invite all the dentists and their families to come south in the delightful month of June, when the roses are all abloom and the flowers flash their colors from every nook and corner. June is not the hot month in this part of Tennessee, so, the invitation is, come, one and all!

---

MICHIGAN STATE DENTAL SOCIETY will meet in Detroit, April 12, 13, 14, 1917. For programme and full particulars write to Clare G. Bates, Durand, Mich.

---

FORSYTH DENTAL LIBRARY AND MUSEUM. The trustees have appointed Dr. Frederick Keyes, Librarian and Curator of the Museum. A strong effort will be put forth at once to collect a complete dental library and odontological museum. Further information may be had by writing to DR. KEYES, in care of The Forsyth Dental Infirmary, 140 The Fenway, Boston.

---

NATIONAL ASSOCIATION OF DENTAL FACULTIES. The 1917 meeting of the N. A. D. F. will be held at the Astor Hotel, New York City, Friday and Saturday, October 19 and 20, 1917.

Executive Committee will meet at 9 a. m. and the general session will open at 10 o'clock as usual.

CHAS. CHANNING ALLEN, D.D.S., *Secy.*
N. W. Cor. Tenth and Troost, Kansas City, Mo.

---

NATIONAL DENTAL ASSOCIATION ANNOUNCEMENTS. The National Dental Association will meet in New York City, October 22-26. The headquarters will be at the Hotel Astor, situated on Broadway, at 44th and 45th streets. This hotel has the largest ball room in the world, and this room will be used for all the general assembly meetings. Other large ball rooms will accommodate the sections, house of delegates, etc. The exhibits will be shown in the beautiful roof gardens. Thus practically all the meetings will occur under the roof of this spacious hotel.

Full accounts of the plans of what promises to be the largest and greatest meeting in the history of the Association will be published later. Suffice it for the present to state that the slogan for this year will be: QUALITY RATHER THAN QUANTITY. Nevertheless there will be quantity also. But the important announcement at this time must be the warning, "*Reserve your rooms at once. Make reservations by mail direct to the hotel of your choice.*" This may seem premature considering the abundance and variety of hotel accommodations listed below. But New York hotels are always crowded. Nearly seven hundred conventions met here during 1916. October is one of the busiest months. If you desire to get into any particular hotel, therefore, it will be safest to write at once. For example, 150 rooms have been reserved at the Headquarters Hotel, The Astor, already.

The following is a list of hotels and rates:

*Hotel Astor, Times Square, 1000 Rooms* (General and Registration Headquarters).—Single with bath, $4.00-6.00; double with bath, $5.00-7.00; two connecting rooms with bath (3 persons), $9.00-11.00; two connecting rooms with bath (4 persons), $10.00-12.00.

*Hotel McAlpin, Broadway and 34th Street, 1600 Rooms.*—Single without bath, $2.50, $3.00; single with bath, $3.00-4.00; double without bath, $3.50, $4.00; double with bath, $4.00-5.00; parlor, bedroom, and bath (for 1 or 2), $6.00-8.00.

*Hotel Waldorf Astoria, Fifth Avenue and 34th Street, 1300 Rooms.*—Single without bath, $3.00; single with bath, $4.00; double without bath, $4.00; double with bath, $6.00; parlor, bedroom, and bath (for 1 or 2), $14.00.

*Hotel Biltmore, Madison Avenue and 43d Street, 1000 Rooms.*—Single with bath, $4.00, $5.00; double with bath, $6.00, $7.00.

*Hotel Manhattan, Madison Avenue and 42d Street, 700 Rooms.*—Single without bath, $2.50; single with bath, $3.50, $4.00; double with bath, $5.00.

*Hotel Albemarle, Broadway and 54th Street.*—Single with bath, $2.00; double with bath, $3.00; parlor, bedroom, and bath (2 persons), $4.00.

*Hotel Ansonia, Broadway and 73d Street, 1600 Rooms.*—Single without bath, $2.00; single with bath, $2.50; double without bath, $3.00; double with bath, $5.00; two connecting rooms with bath, $6.00; three connecting rooms with bath, $10.00.

*Hotel Algonquin, 59 West 44th Street, 250 Rooms.*—Single with bath, $2.50; double with bath, $3.50; double bedroom, sitting room, and bath, $5.00, $6.00.

*Hotel Woodstock, 43d Street near Broadway, 365 Rooms.*—Single without bath, $2.00; single with bath, $2.50; double without bath, $3.00; double with bath, $3.50; two double with bath, $8.00; two single with bath, $5.00; double and single (for 3), $5.50; sitting room, bedroom, and bath (for 1 or 2), $7.00.

*Hotel Woodward, Broadway and 55th Street, 400 Rooms.*—Single without bath, $1.50; single with bath, $2.00; double with bath, $3.00; two connecting rooms with bath (for 2 or 3), $4.00.

A copy of the full list of hotels and rates in pamphlet form will be sent to any member of the National Dental Association on application. Please inclose stamp.

R. OTTOLENGUI, *Chairman Publicity Committee,*

80 West 40th Street, New York City.

